# Identifying the sources of the pulse artefact in EEG recordings made inside an MR scanner

**DOI:** 10.1016/j.neuroimage.2012.12.070

**Published:** 2013-05-01

**Authors:** Karen J. Mullinger, Jade Havenhand, Richard Bowtell

**Affiliations:** Sir Peter Mansfield Magnetic Resonance Centre, School of Physics and Astronomy, University of Nottingham, University Park, Nottingham, NG7 2RD, UK

**Keywords:** Simultaneous EEG–fMRI, Pulse artefact, Artefact reduction

## Abstract

EEG recordings made during concurrent fMRI are confounded by the pulse artefact (PA), which although smaller than the gradient artefact is often more problematic because of its variability over multiple cardiac cycles. A better understanding of the PA is needed in order to generate improved methods for reducing its effect in EEG–fMRI experiments. Here we performed a study aimed at identifying the relative contributions of three putative sources of the PA (cardiac-pulse-driven head rotation, the Hall effect due to pulsatile blood flow and pulse-driven expansion of the scalp) to its amplitude and variability. EEG recordings were made from 6 subjects lying in a 3 T scanner. Accelerometers were fixed on the forehead and temple to monitor head motion. A bite-bar and vacuum cushion were used to restrain the head, thus greatly attenuating the contribution of cardiac-driven head rotation to the PA, while an insulating layer placed between the head and the EEG electrodes was used to eliminate the Hall voltage contribution. Using the root mean square (RMS) amplitude of the PA averaged over leads and time as a measure of the PA amplitude, we found that head restraint and insulating layer reduced the PA by 61% and 42%, respectively, when compared with the PA induced with the subject relaxed, indicating that cardiac-pulse-driven head rotation is the dominant source of the PA. With both the insulating layer and head restraint in place, the PA was reduced in RMS amplitude by 78% compared with the relaxed condition, the remaining PA contribution resulting from scalp expansion or residual head motion. The variance of the PA across cardiac cycles was more strongly reduced by the insulating layer than the head restraint, indicating that the flow-induced Hall voltage makes a larger contribution than pulse-driven head rotation to the variability of the PA.

## Highlights

► We experimentally isolate the putative sources of the pulse artefact (PA) in humans. ► Head rotation is the dominant source its removal produces a 61% reduction of the PA. ► Removing Hall effect produces a considerable, 42%, reduction in PA. ► Hall effect produces largest contribution to PA variance across cardiac cycles.

## Introduction

Simultaneous electroencephalography (EEG) and functional magnetic resonance imaging (fMRI) is becoming a widely used technique for studying brain function. Over the last decade the applications of this technique have grown rapidly as new methods for improving data quality have been developed. Current applications include the study of resting state brain networks (Laufs et al., 2003) and the correlation of natural variations in externally stimulated neuronal responses measured using EEG and fMRI (Benar et al., 2007; Debener et al., 2006; Eichele et al., 2005). To date, the most widely explored clinical application is the non-invasive identification of epileptic foci (Salek-Haddadi et al., 2002, 2003). Recently, the multi-modal EEG–fMRI technique has also been used to investigate sleep (Czisch et al., 2002; Stern et al., 2011) and has been shown to have potential uses in the study of sleep disorders (Ritter and Villringer, 2006). Despite the many examples of the successful application of simultaneous EEG–fMRI in the neurosciences, current investigations are still generally limited by the reduction in the quality of EEG data that results when measurements are acquired during concurrent fMRI.

This reduction in data quality results from the production of artefact voltages in EEG data. The two main artefacts are the gradient artefact (GA) and the pulse artefact (PA). The GA is caused by the time-varying magnetic field gradients that are used for spatial encoding in MRI. The process by which this artefact is generated is well understood and the predictable and periodic nature of the GA, means that the use of average artefact subtraction (AAS) (Allen et al., 1998) or variations thereof (e.g. (Moosmann et al., 2009)) can largely eliminate this artefact in post-processing.

The pulse artefact, which is linked to the cardiac cycle, is less well understood and significantly less predictable in nature than the GA. In particular, the PA shows considerable differences when compared across subjects (Huang-Hellinger et al., 1995) and can also vary in form across cardiac cycles in an individual subject (Bonmassar et al., 2002; Debener et al., 2007). The periodic nature of the PA means that it is also amenable to correction using AAS, but variation of the artefact across cardiac cycles reduces the efficacy that can be achieved in PA correction via AAS, compared with the performance that can be achieved in correcting the GA. The variation of the PA waveform over time means that the artefact template used in AAS is generally formed by averaging over a small number of cardiac cycles. However, if the averaging is done over too few cycles then neuronal signals of interest may also be attenuated in the correction procedure; as a compromise, a sliding window template based on the average of around ten repetitions of the cardiac cycle (Allen et al., 1998) is therefore typically used for PA correction via AAS. Given the limitations of using AAS for PA correction, it is not surprising that significant effort has been dedicated to devising improved techniques for PA correction. Much of this effort has been focused on blind source separation methods, such as independent component analysis (ICA) (Briselli et al., 2006; Mantini et al., 2007) and optimal basis set (OBS) analysis (Debener et al., 2007; Naizy et al., 2005). Spatial filtering of the EEG measurements, for example via use of spatially adaptive beamformer methods (Brookes et al., 2008), has also been applied to PA removal.

The reported level of success resulting from the use of these artefact correction methods varies considerably between studies, with greater efficacy of correction generally achieved at lower field strengths. This field strength dependence is not surprising, given it is known the amplitude of the PA scales with field strength (Debener et al., 2008). For example, in early EEG–fMRI experiments carried out at 1.5 T, Huang-Hellinger et al. (1995) reported that alpha oscillations could be observed above the PA amplitude in some subjects. However, the peak amplitude of the PA at 3 T can exceed 200 μV (Debener et al., 2007), thus swamping the scalp voltages resulting from brain activity. The increase in signal- and contrast-to-noise-ratio with magnetic field strength in fMRI data provides a strong motivation for moving to higher field strengths, such as 7 T, for fMRI data acquisition (Gati et al., 1997; van der Zwaag et al., 2009) especially when studying the trial-by-trial variability of the response to a stimulus. However, the greater degradation of the EEG data at ultra-high field due to the larger residual PA (Debener et al., 2008) means that moving to higher field may not always be beneficial in simultaneous EEG–fMRI studies. In order to realise the full benefit of high field in EEG–fMRI, better efficacy in removal of the PA is therefore needed and attainment of this goal requires an improved understanding of the source of this artefact and its variability over cardiac cycles.

A number of putative mechanisms by which cardiac pulsation could generate the PA have previously been proposed (Allen et al., 1998; Debener et al., 2008). As discussed by Yan et al. (2010), the most plausible of these are: (i) voltages induced by cardiac-pulse-driven rotation of the head in the strong static magnetic field of the MR scanner ‒ this rotation is driven by changes in the momentum of blood as it is shunted into arteries in the head (Bonmassar et al., 2002; Huang-Hellinger et al., 1995); (ii) the pulsatile flow of blood, which is an electrically conducting fluid, in the presence of a magnetic field ‒ this flow produces a separation of charge via the Hall effect (Tenforde et al., 1983) that in turn gives rise to voltage variation at the surface of the scalp (Muri et al., 1998); and (iii) voltages generated by movement associated with pulse driven expansion of the scalp (Debener et al., 2008). A better understanding of the relative contributions of each of these potential sources of artefact would be valuable for the development of new methods for reducing the PA either at source or via novel correction methods. The magnitude and spatial variation of the artefact voltages that are expected to result from cardiac-pulse-driven head rotation and the Hall effect, due to blood flow in the main arteries of the brain, have recently been evaluated using simple models (Yan et al., 2010). This work demonstrated that both cardiac-pulse-induced head nodding and pulsatile blood flow in brain arteries running in an anterior–posterior direction could produce artefacts with a spatial topography similar to that of the experimentally observed PA. However, the results also showed that, while realistic rates of pulse-driven head rotation could produce artefact voltages consistent with the measured PA, typical pulsatile variations in blood velocity in large brain arteries would produce artefact voltages that are much smaller than those measured experimentally. The conclusions drawn from this modelling work were that the main source of the PA is most likely to be pulse-driven head rotation, but that both pulsatile blood flow in superficial arteries and scalp expansion may also contribute to the complex PA waveform. This inference is consistent with previous experimental work (Anami et al., 2002) in which head fixation through the use of a vacuum cushion was shown to produce a large reduction of the amplitude of the PA in two subjects.

Here, we describe an experimental investigation of the causes of the PA, based on in vivo recordings from healthy volunteers. By isolating the effects of each of the putative sources of the PA we aim to assess their relative contributions to the average artefact and its variation over multiple cardiac cycles. Simultaneous recording of the signals from piezoelectric accelerometers attached to the head also allowed investigation of the link between head movements and the PA.

## Methods

### Acquisition

EEG recordings were made inside a 3 T scanner on 6 subjects (age = 26 ± 3 yrs, 4 males) with no history of neurological or cardiac disorders. The study was carried out with approval of the local ethics committee and informed consent was obtained from each subject. EEG data were acquired with a 32-channel EEG cap, incorporating 31 electrodes following the extended international 10–20 system and a reference electrode, positioned at FCz. Electrode impedances were kept below 20 kΩ. The cap contained an additional lead for electrooculography (EOG) which was attached beneath the left eye, but data from this channel were not employed in the PA analysis. A BrainAmp MR-plus EEG amplifier (Brain Products, Gilching, Germany) was used to record the EEG data. Data were band-pass filtered by the amplifier hardware to the frequency range, 0.016–250 Hz, which is commonly used in simultaneous EEG–fMRI experiments. Head movement was monitored using two, MR-conditional, 3-axis accelerometers (Brain Products, Gilching, Germany), which provided simultaneous measurements of the acceleration along three orthogonal directions. These were fixed onto the forehead (between electrodes Fp1&Fp2) and the right temple (above electrode T8) using a combination of Micropore tape and a loop of elastic fabric which was adjusted to the size of each individual's head. The cardiac trace was recorded using two electrocardiogram (ECG) electrodes placed on the chest. Signals from the ECG electrodes and the accelerometers were recorded using a BrainAmp ExG-MR bipolar amplifier. All data were recorded simultaneously using Vision Recorder (version 1.10).

Four minutes of data was acquired from each subject in four different conditions:

i) Relaxed, yielding a PA that is typical for EEG–fMRI;
ii) With the head restrained through use of a bite-bar and evacuated vacuum cushion;
iii) With a thin insulating layer placed between the scalp and the EEG cap, and an outer layer of conducting gel then used to form connections between the electrodes;
iv) With the head restrained using the bite-bar and evacuated vacuum cushion, and with the insulating layer also in place ‒ i.e. (ii) and (iii) combined.

The bite-bar and vacuum cushion were used in conditions (ii) and (iv) to reduce pulse-driven head rotation. A dental impression was made for each subject prior to scanning, using impression trays (Orthocare, UK) and impression compound (Kerr, Italy). The impression was then attached to the bite-bar, which was in turn secured to the MR scanner bed. The position of the bite-bar was adjusted to maximise subject comfort. The vacuum cushion was placed under the subject's head and wrapped around the sides of the head before evacuation. In conditions (iii) and (iv) a silicone rubber swimming cap was used to isolate the EEG electrodes from Hall voltages produced by blood flow. A layer of Abralyte 2000 (Brain Products, Gilching, Germany) gel was smeared over the exterior of the swimming cap before the EEG cap was put in place, yielding electrode impedances of less than 15 kΩ. We consequently assume that in condition (ii) artefact voltages due to pulse-driven head rotation are largely eliminated leaving only the PA contributions due to flow-induced Hall voltages and pulse-driven scalp expansion, while in condition (iii) Hall voltages are eliminated leaving contributions of pulse-driven head rotation and scalp expansion. In condition (iv), both Hall voltages and head rotation effects are largely eliminated, leaving just the effect of pulse-driven scalp expansion.

All recordings from an individual subject were made in the same session so as to minimise possible confounding effects on the PA of differences in cardiac activity due to varying external influences, such as previous physical exercise or intake of caffeine. Due to practical limitations in the set-up procedure, data were acquired from all subjects in the order in which the conditions are listed. Subjects lay inside the 3 T magnet of a Philips Achieva MR scanner (Philips Medical Systems, Best, Netherlands), with the head positioned on a head-rest located within the homogeneous region at the centre of the magnet, as would be the case for a standard fMRI experiment. However, no MR scanning was carried out, so that the PA could be recorded without the confounding effect of the gradient artefact. Subjects were asked to lie still with their eyes open during recording.

Data were also recorded outside the scanner from two subjects (drawn from the cohort used in the main study) in order to assess the contribution of low, frequency resting state brain activity (Orrison, 1995) to the measurements made when the insulating layer was not in place. Two recordings were made: one with the subject in the relaxed condition and a second where the insulating layer was used. For both recordings the subjects lay in a supine position and the conditions were generally made as similar as possible to those occurring inside the scanner.

An additional experiment was carried out to allow estimation of the signal variation due to the elevated electromagnetic noise inside the scanner. To assess the magnitude of this noise, recordings were made with the EEG cap fitted onto a spherical agar phantom, with similar conductivity to tissue (as described in (Yan et al., 2009)), which was placed inside the 3T scanner.

### Analysis

Initial analysis was carried out using Analyzer2 (v2.0.1, Brain Products). R-peak markers were added to the data by applying an automatic peak detection method to the ECG trace. Based on visual inspection of the data, any incorrectly placed markers were manually moved to the relevant R-peak position.

Data were then filtered to the 0.1–20 Hz frequency range, so as to remove muscle artefacts and other high frequency noise, before being exported for further analysis using Matlab (Mathworks, UK). The EEG and accelerometer recordings were then segmented using the R-peak markers (− 100 ms to + 600 ms in extent relative to R-peak) and baseline-corrected. The EEG data were corrected based on the 100 ms of data recorded before each R-peak (− 100 ms to 0). The accelerometer data were baseline corrected relative to the mean of the whole segment so as to remove drift, before being integrated over time to give a measure of velocity for each of the three orthogonal channels in the two motion sensors. In measurements made with the accelerometers in a stationary state we found that the standard deviation of the velocity measure due to noise in the accelerometer signals was 0.07 mm/s.

To form a measure of the average PA for each subject in each condition, the mean PA waveform on each channel was calculated by averaging over the first 90 cardiac cycles producing clear R-peaks in the ECG trace. The root-mean-square (RMS) amplitude over channels was then calculated at each time point, along with the RMS over time points in the average waveform on each channel. The difference of the maximum and minimum voltages in the average PA was also calculated for each channel and the largest value across channels used as a measure of the range of the artefact amplitude for each subject and condition. The mean and standard deviation of the artefact range over subjects were also calculated.

A group measure for each condition was created by averaging the RMS waveforms over all subjects. The associated standard deviation over subjects was also calculated at each time point. The mean RMS amplitude over a cardiac cycle was also calculated and the average over subjects used as a useful, single measure of the PA magnitude in each condition. Similar measures of the velocity variation were calculated, in which the RMS over the three orthogonal velocity channels at each time point was used as an overall measure of motion at the two different sensor locations. The standard deviation of the PA over the 90 cardiac cycles was also calculated at each time-point and then averaged over channels and subjects, to provide a measure of the variability of the PA across heart-beats in each condition. Since the data were baseline corrected using the 100 ms of data preceding each R-peak, the variance was greatly reduced in this time window. Consequently the standard deviation was averaged over the time window from 50 to 600 ms after the R-peak to provide a single a measure of the variability of the PA across cardiac cycles in each condition. Wilcoxon signed-rank tests were used to assess the significance of differences in the amplitude and variance of the PA and velocity measures between the different conditions. The RMS and standard deviation of the PA in each condition were compared with the values measured in the relaxed condition and characterised by using the percentage reduction compared with the relaxed condition (i.e. comparing to the PA which would be present in standard EEG–fMRI experiments).

The recordings on the phantom were analysed by initially placing artificial R-peak markers in the data using timings taken from one of the subject's recordings. These data and the data acquired on human subjects outside the scanner were then analysed using identical methods to those described above, so as to allow assessment of the contributions of brain signals and electromagnetic noise to the measurements.

## Results

**Fig. 1:**
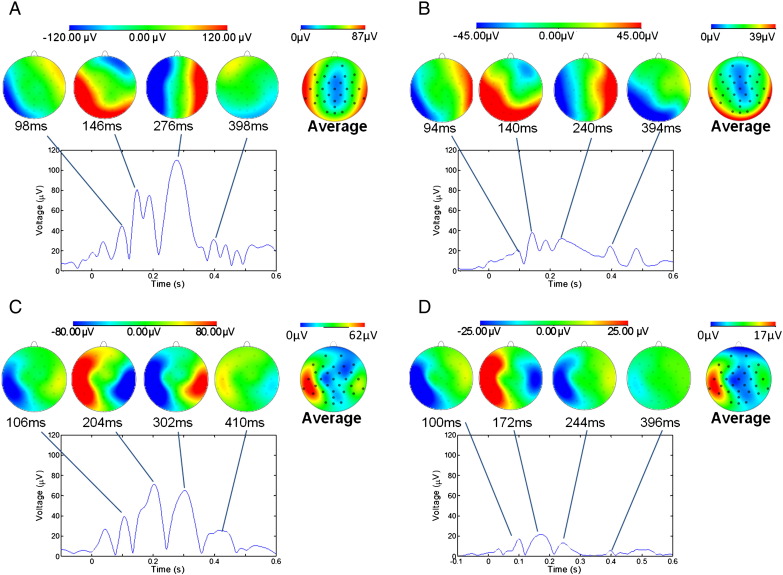
The RMS over channels of the average pulse artefact for a representative subject in the four different conditions: A) relaxed; B) restrained; C) EEG cap insulated from the scalp; and D) restrained and insulated. Voltage maps, scaled to absolute maximum RMS values, are shown for four different latencies, corresponding to the peaks in the RMS waveform for each condition. The average maps for each condition are maps of the RMS (over time) of the average pulse artefact.

**Fig. 2:**
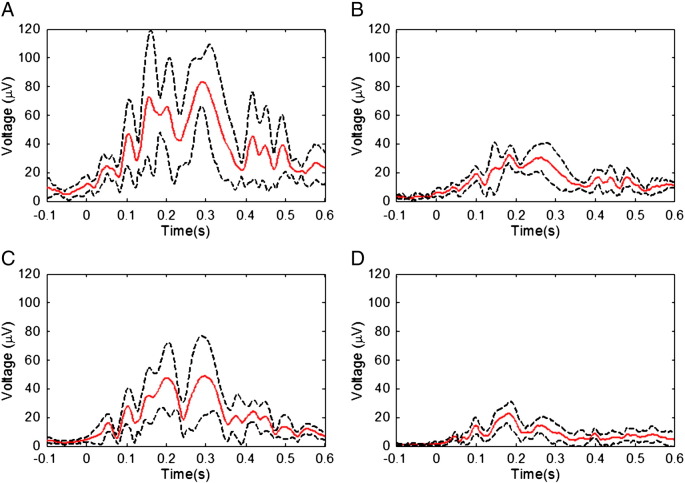
The RMS over channels of the cycle-average PA after averaging over subjects for the four different conditions: A) relaxed; B) restrained; C) EEG cap insulated from the scalp; and D) restrained and insulated. The average (red line) and standard deviation (dashed lines) over subjects are shown.

**Fig. 3:**
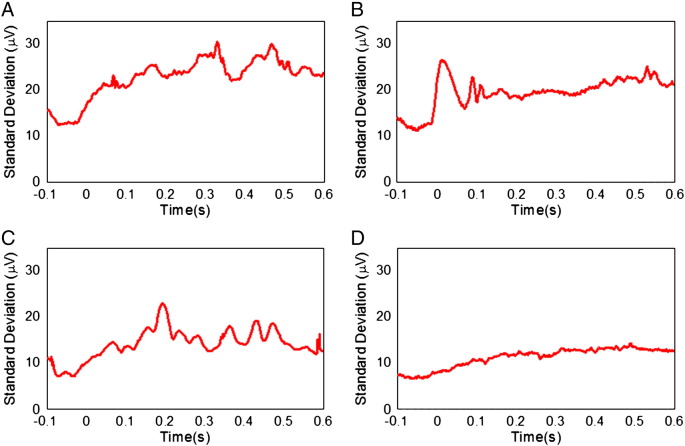
The standard deviation of the pulse artefact across cardiac cycles, averaged over subjects for the four different conditions: A) relaxed; B) restrained; C) EEG cap insulated from the scalp; and D) restrained and insulated.

Fig. 1 shows the temporal variation of the RMS amplitude over leads of the average PA recorded from a representative subject in the four different conditions. The spatial maps depict the scalp topography of the artefact at peaks in the RMS traces. In addition, the average maps show the spatial variation of the RMS amplitude of the average PA over the cardiac cycle. Table 1 details the largest range of the induced artefact over all channels in the different conditions, while Fig. 2 shows the average and standard deviation over subjects of the RMS of the artefact. The average waveforms in Fig. 2 display similar characteristics to the single subject data shown in Fig. 1. In particular, the peaks highlighted in Fig. 1 are also easily identifiable in Fig. 2. The two main peaks of the PA can be seen to occur at approximately 170 and 260 ms relative to the R-peak when the subject is relaxed (Fig. 2A), in agreement with previous findings (Debener et al., 2008; Yan et al., 2010). The dotted lines in Fig. 2, which characterise the standard deviation of the PA across subjects, indicate that the variability is largest around the main peaks of the artefact. The low value of the standard deviation over the time range − 100 to 0 ms is a consequence of the baseline correction process applied to each cardiac cycle. Fig. 3 shows the average over subjects and leads of the standard deviation of the PA across cardiac cycles, indicating the level of artefact variability across cycles, which was present in each condition.

**Fig. 4:**
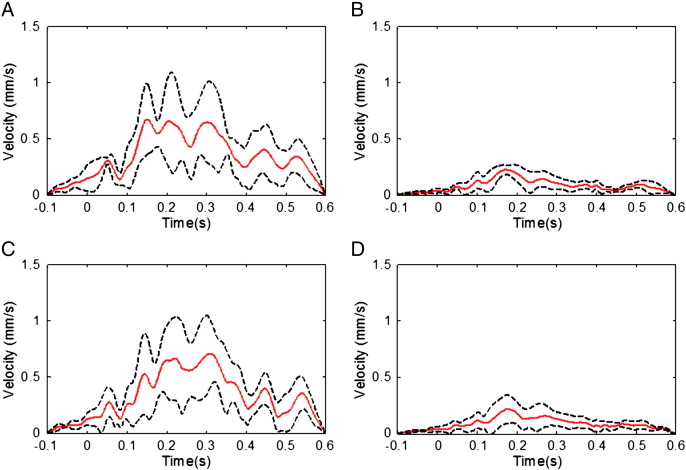
The RMS of the velocity measures derived from the orthogonal channels of the accelerometer placed on the forehead, for the four different conditions: A) relaxed; B) restrained; C) EEG cap insulated from the scalp; and D) restrained and insulated. The average (red line) and standard deviation (dashed lines) over subjects are shown.

Fig. 4 shows the temporal variation of the average RMS velocity, measured from the accelerometer positioned on the forehead, over a cardiac cycle. Comparison of Figs. 2 and 4 indicates that the RMS velocities are largest in periods when the largest RMS artefact voltages occur in all of the different conditions. Fig. 4 also shows that some residual movement is recorded at the forehead even when the subjects' head movements are restrained by the use of the bite-bar and evacuated vacuum cushion (Figs. 4B & D). The dashed lines in Fig. 4 indicate the standard deviation of the RMS velocity measure over subjects.

**Fig. 5:**
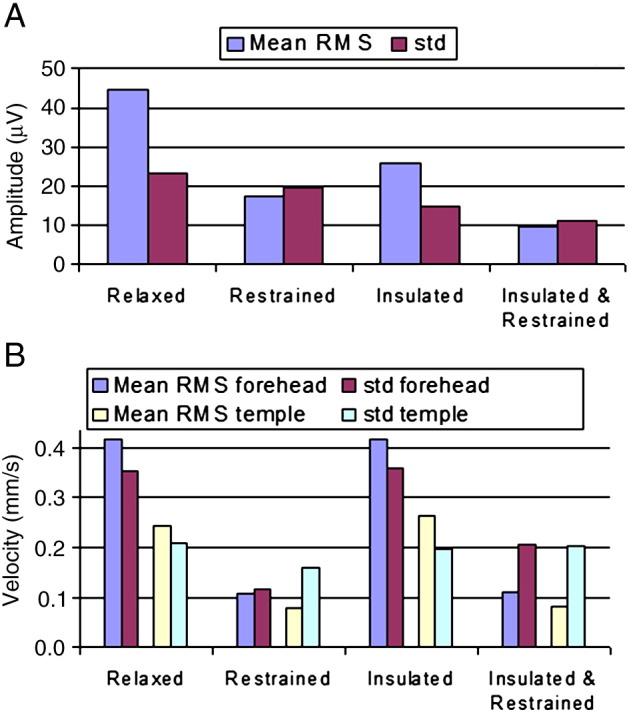
The RMS and standard deviation in each condition averaged over subjects and cardiac cycle for: A) the EEG artefact; and B) and head motion (also see Supplementary Table). The RMS and standard deviation in each condition averaged over subjects and cardiac cycle for: A) the EEG artefact; and B) and head motion (also see Supplementary Table).

Fig. 5 compares the mean RMS and standard deviation of the PA and velocity measures which were formed by averaging over the cardiac cycle time courses shown in Figs. 2–4. These data (also tabulated in Supplementary Material) show that, when compared with the same measures during the relaxed condition, a significant (P < 0.03), 61%, reduction in the mean RMS amplitude of the PA was produced by restraining the movement of the head using the bite-bar and vacuum cushion (Figs. 2B & 5A), while the RMS velocity recorded on the forehead was decreased by 74% (P < 0.03) by this head restraint (Figs. 4B & 5B). While the reduction in the standard deviation of the velocity measured on the forehead resulting from head restraint was similar to the reduction in RMS velocity amplitude (67%, P < 0.03), the reduction in the standard deviation of the PA was much smaller (16%, P < 0.05) than the reduction in the amplitude of the PA. Use of the insulating layer also produced a significant, 42% decrease (P < 0.05) in the PA amplitude (Figs. 2C & 5A) and also reduced the standard deviation of the PA by 37% (P < 0.05). As would be expected, the measured head motion was not significantly changed (Figs. 4C & 5B) by the presence of the insulating layer. Combined use of the head restraint and insulating layer produced the greatest reduction in the RMS and standard deviation of the PA (78%, P < 0.03 and 52%, P < 0.05 respectively) compared with the relaxed condition, with the RMS and standard deviation of the velocity measured on the forehead also being reduced by comparable amounts (73%, P < 0.03 and 42%, P < 0.05 respectively). The RMS velocity measured by the accelerometer placed on the temple was similarly reduced when head movement was constrained (67% reduction when head restrained, no significant change in presence of the insulating layer and a 66% decrease with the combination of the head restraint and insulating layer), but there was no significant change in the standard deviation of this measurement across cardiac cycles for any of the conditions.

From the measurements acquired outside the scanner, the mean standard deviation across cardiac cycles was found to be reduced from 9.3 μV to 0.5 μV when voltages due to brain activity were blocked by the insulating layer. This suggests that a significant proportion of the reduction in the standard deviation of the PA that was produced by the insulating layer was due to the removal of the effects of brain activity. The recording on the phantom inside the MR scanner yielded a noise-related standard deviation of 6.2 μV.

## Discussion

Figs. 1, 2 and 5 indicate that the RMS amplitude of the PA is significantly attenuated when head movement is reduced through the use of a bite-bar and vacuum cushion and also attenuated, but by a lesser degree, when an insulating layer is placed between the electrodes and the scalp. The maximum reduction of the RMS amplitude of the PA occurs when the head restraint and insulating layer are both employed. We also found that the reductions of the largest range of the induced artefact over channels (Table 1) that resulted from use of the head restraint and insulating layer, separately and together followed a similar pattern to that seen for the RMS amplitude (Fig. 5).

The spatial pattern of the PA at the temporal peaks remains similar in the different conditions, showing a predominantly left to right variation (Debener et al., 2008; Yan et al., 2010), even though the amplitude of the artefact is significantly attenuated by the use of the head restraint and insulating layer (Fig. 1). The maps showing the spatial variation over leads of the RMS amplitude of the average PA across a cardiac cycle indicate that the largest values occur in lateral regions in the relaxed condition. The map obtained in the restrained condition shows a more uniform spread of elevated amplitude around the lateral and posterior regions, but with reduced amplitude. When the insulating layer is put in place the RMS amplitude of the artefact is lateralised again and is more specific to the temple regions. These results may suggest that the Hall effect induces artefacts more uniformly around the edge of the head (where the distance from the reference electrode is largest) whereas the head rotation induces artefacts which are more lateralised.

The measurements made using the accelerometers placed on the forehead and temple (Figs. 4 and 5) show that pulse-driven head movements are significantly reduced by the use of the bite-bar and vacuum cushion, but are unaffected by the presence of the insulating layer. The larger reduction of the PA which we measured when using the head restraint (27 μV reduction in the RMS amplitude) compared with the insulating layer (19 μV reduction), evident from Figs. 2 & 5, is therefore consistent with the hypothesis that cardiac-pulse-driven head rotation is the dominant source of the PA (Anami et al., 2002; Yan et al., 2010). Although it is difficult to make detailed quantitative comparisons between the velocities of movement measured at the temple and the forehead because of the different sensor orientations and the interaction of the effects of gravity and rotation on the accelerometers, the much larger velocity measured at the forehead is consistent with the suggestion that nodding (corresponding to the motion known as “pitch”) is the dominant cardiac-driven head motion (Yan et al., 2010).

The smaller reduction of the PA produced by the insulating layer alone could be explained by there being a contribution to the PA from flow-induced Hall voltages that is eliminated when the conducting paths from the vessels to the electrodes are blocked by the insulating layer. However, we cannot rule out the possibility that this attenuation of the PA could have resulted from a reduction of the head-motion-induced voltages due to the alteration of the current paths in the moving conductor, formed by the head, when the insulating layer is put in place. Specifically, when the insulating layer is used, currents between electrodes are confined to the thin conducting layer formed by the gel on the surface of the swim cap rather than spreading throughout the head. Stronger evidence for the existence of a significant Hall voltage contribution to the PA is however provided by the additional (8 μV) reduction of the RMS PA amplitude that was found when the insulating layer and head restraint were used together, compared to the use of head restraint only. To support this assertion, we first note again that the measured head motion is reduced by a factor of 4 in the restrained condition (Fig. 5). Second, the reduction in RMS PA amplitude on going from the relaxed to insulated case is 19 μV. If we assume that the reduction in the latter case is entirely due to a change in the motion induced voltages when the insulating layer is applied (as a result in the change in current paths in the volume conductor) then the reduction from addition of the insulating layer would be expected to be 4 times smaller (i.e. ~ 4.7 μV) when the head is restrained. Taking these two factors together, it seems unlikely that the 8 μV reduction in the mean RMS amplitude of the PA which occurs on addition of the insulating layer when the head is restrained is due to a change in the voltage induced by any residual head motion. It is however consistent with the hypothesis that there is a significant Hall voltage contribution to the PA which is eliminated by the insulating layer.

Such a Hall voltage contribution to the PA is considerably larger than was predicted in previous modelling work (Yan et al., 2010), which considered the effect of pulsatile blood flow in the large arteries in the brain. It is likely therefore that the dominant Hall voltage contribution to the PA is actually produced by vessels in the scalp. Although when measured at the vessel wall, the fluctuating Hall voltages due to pulsatile blood flow in small arteries in the scalp will be smaller than those produced in the large arteries in the brain, scalp vessels may produce the larger artefact voltages in EEG recordings because of their closer proximity to the electrodes and the fact that the voltages which they generate at these electrodes are not attenuated by the poorly conducting skull.

Before making categorical attributions of the measured artefact voltages to the effects of head rotation and flow-induced Hall voltages, we should also consider the possibility of there being differences in the contribution of pulse-driven scalp expansion to the PA in the four different conditions. The use of the bite-bar clearly should not affect scalp expansion whilst the positioning of the vacuum cushion also meant that it was unlikely to moderate any scalp pulsation. However, since the insulating layer consisted of a tight-fitting swim-cap it is possible that its presence reduced any pulse-driven scalp expansion. To assess this effect, we used a tight fitting bandage placed over the EEG cap to apply a similar level of compression to the scalp as was produced by the swim-cap. We recorded the PA generated inside the scanner with and without this bandage applied (without the insulating layer or head restraint) to five of the subjects previously studied, and found that across the group the bandage had no effect on the amplitude or standard deviation of the PA. It can therefore be concluded that the scalp compression produced by the swim cap had negligible effect on the measured PA, suggesting that this compression was not significant enough to affect the pulse-driven scalp expansion or that the contribution of scalp expansion to the PA is negligible. The reduction of the PA due to addition of the insulating layer with the head restrained can therefore be taken as strong evidence for a flow-related Hall voltage contribution to the PA.

The combined use of the head restraint and insulating layer still left a residual PA (Fig. 2D) of about 10 μV in RMS amplitude (Fig. 5A) which could be due to (i) residual pulse-driven head rotation or (ii) pulse-driven scalp expansion. It is difficult to discriminate categorically between these two possibilities. The similarity of the spatial topography of the voltages at the peaks of the RMS artefact plots in the relaxed (Fig. 1A) and in the restrained and insulated (Fig. 1D) conditions might suggest (i) since we know that the effect of head rotation dominates in the relaxed condition. However it is possible that scalp expansion and head nodding could produce artefacts of similar topography (Debener et al., 2008; Yan et al., 2010). Since cardiac-driven head rotation is small in magnitude even when the head is unrestrained and the bite-bar severely limits rotation of the head we favour the explanation that this residual PA is a consequence of scalp expansion.

Some further information about the origin of the PA can be gathered by considering the temporal form of the artefact waveform. Fig. 1 shows that the latencies of the main PA peaks measured relative to the R-peak vary between conditions. The latencies of the peaks corresponding to those highlighted in Fig. 1 were identified for all other subjects. A two-way repeated measures ANOVA (factors: condition × peak number) was performed to test for a significant effect of either condition or peak number on latency and also for a possible interaction between the two variables. Similar variation over conditions was observed in all subjects, as is evident from the average RMS waveforms shown in Fig. 2, with the main finding being that the latency of the peaks decreased when the head was restrained, or restrained and insulated, compared with the relaxed condition (P < 0.005 and P < 0.001, repeated measures ANOVA). There was no clear pattern in the variation of the peak latencies when comparing the insulated and relaxed conditions and no significant difference between the peak latencies in the restrained versus the restrained and insulated conditions. Since it was not possible to counterbalance the order of the experiments between subjects these differences in latencies could be due to an ordering effect. A significant difference in heart rate was found between the relaxed and insulated conditions (P < 0.002) however, no significant difference was seen between the relaxed and restrained or relaxed and insulated plus restrained conditions. The insulated condition was not the last recording made and the effects observed on the heart rate do not correlate with the variation in latency of the PA peaks. Therefore, we do not believe that the ordering of the conditions had a significant effect on the results we obtained in this study.

The finding that the most significant change in peak latency occurs when head restraint is applied is consistent with the suggestion that cardiac-driven-head rotation is the dominant source of the PA (Yan et al., 2010; Anami et al., 2002), while the earlier occurrence of the artefact peaks when the head is restrained indicates that changes in both the Hall voltage due to blood flow variation and the artefact voltage produced by scalp expansion precede the voltage changes generated by cardiac-pulse-induced head rotation through the cardiac cycle. This makes sense, since the Hall voltage will instantaneously follow changes in blood flow, while the inertia of the head means that there is likely to be some delay before the changes in blood flow and consequent momentum transfer are translated into head rotation. It is also plausible that scalp expansion precedes head rotation because of the smaller momentum transfer required to accelerate the tissue above a vessel.

Although understanding the source of the PA is important for efforts to reduce the confounding effect of the PA in combined EEG–fMRI experiments, understanding the causes of variability in the artefact across cardiac cycles could be even more valuable, since it is largely this variation of the PA which causes artefact correction methods to fail. The measurements of the standard deviation of the PA across cardiac cycles in the different conditions (Figs. 3 and 5) provide some insight into this variability. These show that the measured standard deviation is reduced more significantly by the insulating layer than the head restraint.

However in order to make a proper comparison we need to consider other sources of variability in the measurements and how these may change across the different conditions. If we appropriately subtract off the variance due to brain activity, which was recorded separately, (i.e. only in the conditions in which the insulating layer is not in place) and also take off the variance due to electromagnetic noise in the scanner so as to pick out the variability of the PA, we find that the average standard deviation across cardiac cycles reduces from 20.3 μV in the relaxed case to 16.1 μV (a 21% reduction) when the head restraint is applied, and reduces to 13.3 μV (a 34% reduction compared with the relaxed case) when the insulating layer alone is applied. This suggests that although the Hall voltage makes a lesser contribution than pulse-driven head rotation to the magnitude of the PA, it is the dominant source of variability in the PA over multiple cardiac cycles. A residual standard deviation of around 10 μV remains when the insulating layer and head restraint are both applied, which is most readily interpreted as meaning that voltages due to pulse-driven scalp expansion are highly variable across cardiac cycles.

Whilst we successfully isolated and attributed the contribution of brain activity and external noise sources to the total variability of the PA, it was not possible to isolate the increased variance which may have occurred in the restrained condition due to muscle activity when the bite-bar was used. However, the stringent low-pass filtering which we employed was designed to remove most muscle activity artefact based on previous description of the frequency spectrum of jaw muscle activity (Van Boxtel, 2001). In addition a comparison of Fourier transforms of the data from the relaxed and restrained conditions revealed no frequency bands in which activity in the restrained condition was greater than that in the relaxed condition. As a result we do not believe that the variance in the restrained condition contained a significant contribution from muscle activity.

The standard deviation over cardiac cycles of the velocity measured at the forehead also shows a significant reduction when the head is restrained (Fig. 5), consistent with the fact that the variability of the PA is also reduced by head restraint, presumably as a result of a reduction in variation of head rotation across cardiac cycles. Although the average standard deviation over cardiac cycles of the velocity measures for the group are smaller for the restrained than the insulated and restrained condition, these differences are not significant (Wilcoxon signed-rank tests) for either the forehead or temple recordings (P < 0.2 and P = 1, respectively). In general the standard deviations of the velocity measurements are comparable in magnitude to the RMS measures of the velocity waveform across conditions, whereas this is only the case for the EEG measurements when the head is restrained or restrained with the insulating layer in place (Fig. 5). This could suggest that there are some significant variations in the local velocity measured at the skin surface as a result of scalp expansion and other skin movements, whose effects on the velocity measurements and EEG artefacts are brought to the fore when rigid body movements are eliminated by the use of the head restraint.

It is important to consider the implications of the findings of this work for dealing with the adverse effects of the PA on EEG recordings made during concurrent fMRI. Clearly an insulating layer cannot be used in real experiments since it also eliminates the brain signals of interest and it is hard to envisage any approach that would allow the Hall voltage contribution to the PA to be attenuated in real EEG–fMRI experiments. The contribution to the PA from head rotation can however be attenuated and our results indicate that use of a head restraint consisting of a bite-bar and vacuum cushion can reduce the magnitude of the PA, from that recorded with the subject relaxed, by around 60%, while also reducing its standard deviation across cardiac cycles by about 20%. Since the PA that is present at fields of 1.5 T and above is generally many times larger than the brain signals of interest (Debener et al., 2008) such reductions would be expected to produce worthwhile improvements in EEG data quality. However, since a bite-bar is not very comfortable and leads to the production of muscle artefacts that would corrupt EEG recordings, it could not realistically be used in many experimental studies. Similar reductions in the amplitude and variability of the head-rotation induced part of the PA could also however be achieved using other approaches, which use reference signals generated from wire loops on the head (Masterton et al., 2007) or from electrodes on a conducting reference layer that is electrically isolated from the scalp (Chowdhury et al., 2012; Dunseath et al., 2009). Since head rotation induces artefact voltages in the reference layer or reference loops as well as in the signals measured from the scalp, the rotation contribution to the PA can potentially be eliminated by subtracting off the reference layer signal from the scalp recordings. This approach may also potentially reduce the PA contribution due to scalp expansion, but will not attenuate the Hall voltage contribution to the PA since this is not seen by the reference loops or layer. Since these reference-signal-based approaches can also greatly attenuate the gradient artefact, they are well worth pursuing.

The following is the supplementary data related to this article.

Supplementary Table The precise values (to 3 significant figures) of the RMS and standard deviation in each condition averaged over subjects and cardiac cycle for the EEG artefact and velocity measured using accelerometers affixed to the forehead and temple (data also shown in Fig. 5).

## Figures and Tables

**Table 1:** The maximum range in the average pulse artefact over all channels averaged over subjects. The error is the standard deviation of the peak to peak measure across subjects.

Condition	Relaxed	Restrained	Insulated	Restrained & insulated
Mean ± standard deviation (μV)	430 ± 80	170 ± 30	240 ± 60	90 ± 20
