# Supplementary material for: Identifying the sources of the pulse artefact in EEG recordings made inside an MR scanner
Source: Neuroimage. 2013 May 1;71:75–83. doi: 10.1016/j.neuroimage.2012.12.070 (PMC3601330; doi:10.1016/j.neuroimage.2012.12.070)
Supplement: Supplementary Table — The precise values (to 3 significant figures) of the RMS and standard deviation in each condition averaged over subjects and cardiac cycle for the EEG artefact and velocity measured using accelerometers affixed to the forehead and temple (data also shown in Fig. 5). [file mmc1.docx]

**Supplementary Table:**

|  | **Relaxed** | | **Restrained** | | **Insulated** | | **Restrained & Insulated** | |
| --- | --- | --- | --- | --- | --- | --- | --- | --- |
|  | RMS | STD | RMS | STD | RMS | STD | RMS | STD |
| **EEG amplitude (μV)** | 44.5 | 23.2 | 17.5 | 19.5 | 25.6 | 14.7 | 9.82 | 11.8 |
| **Forehead velocity (mm/s)** | 0.416 | 0.352 | 0.107 | 0.116 | 0.416 | 0.359 | 0.111 | 0.205 |
| **Temple velocity (mm/s)** | 0.242 | 0.210 | 0.080 | 0.160 | 0.262 | 0.197 | 0.082 | 0.202 |

**Supplementary Table:** The precise values (to 3 significant figures) of the RMS and standard deviation in each condition averaged over subjects and cardiac cycle for the EEG artefact and velocity measured using accelerometers affixed to the forehead and temple (data also shown in Figure 5).
